# Characterization of Reemerging Chikungunya Virus

**DOI:** 10.1371/journal.ppat.0030089

**Published:** 2007-06-29

**Authors:** Marion Sourisseau, Clémentine Schilte, Nicoletta Casartelli, Céline Trouillet, Florence Guivel-Benhassine, Dominika Rudnicka, Nathalie Sol-Foulon, Karin Le Roux, Marie-Christine Prevost, Hafida Fsihi, Marie-Pascale Frenkiel, Fabien Blanchet, Philippe V Afonso, Pierre-Emmanuel Ceccaldi, Simona Ozden, Antoine Gessain, Isabelle Schuffenecker, Bruno Verhasselt, Alessia Zamborlini, Ali Saïb, Felix A Rey, Fernando Arenzana-Seisdedos, Philippe Desprès, Alain Michault, Matthew L Albert, Olivier Schwartz

**Affiliations:** 1 Department of Virology, Unité Virus et Immunité, Institut Pasteur, Paris, France; 2 CNRS URA 3015, Paris, France; 3 Department of Immunology, Groupe Immunobiologie des Cellules Dendritiques, Institut Pasteur, Paris, France; 4 INSERM U818, Paris, France; 5 Laboratoire de Microbiologie, Groupe Hospitalier Sud Réunion, Ile de la Réunion, France; 6 Département de Biologie Cellulaire et Infection, Plateforme de Microscopie Électronique, Institut Pasteur, Paris, France; 7 Département Infection et Epidémiologie, Institut Pasteur, Paris, France; 8 Department of Virology, Unité Interactions Moléculaires Flavivirus-Hôtes, Institut Pasteur, Paris, France; 9 Department of Virology, Unité d'Épidémiologie et Physiopathologie des Virus Oncogènes, Institut Pasteur, Paris, France; 10 Centre National de Référence des Arbovirus, Lyon, France; 11 Ghent University, Ghent, Belgium; 12 Hôpital St-Louis, Paris, France; 13 Department of Virology, Unité de Virologie Structurale, Institut Pasteur, Paris, France; 14 Department of Virology, Laboratoire de Pathogénie Virale Moléculaire, Institut Pasteur, Paris, France; The Scripps Research Institute, United States of America

## Abstract

An unprecedented epidemic of chikungunya virus (CHIKV) infection recently started in countries of the Indian Ocean area, causing an acute and painful syndrome with strong fever, asthenia, skin rash, polyarthritis, and lethal cases of encephalitis. The basis for chikungunya disease and the tropism of CHIKV remain unknown. Here, we describe the replication characteristics of recent clinical CHIKV strains. Human epithelial and endothelial cells, primary fibroblasts and, to a lesser extent, monocyte-derived macrophages, were susceptible to infection and allowed viral production. In contrast, CHIKV did not replicate in lymphoid and monocytoid cell lines, primary lymphocytes and monocytes, or monocyte-derived dendritic cells. CHIKV replication was cytopathic and associated with an induction of apoptosis in infected cells. Chloroquine, bafilomycin-A1, and short hairpin RNAs against dynamin-2 inhibited viral production, indicating that viral entry occurs through pH-dependent endocytosis. CHIKV was highly sensitive to the antiviral activity of type I and II interferons. These results provide a general insight into the interaction between CHIKV and its mammalian host.

## Author Summary

Chikungunya virus (CHIKV) is a reemerging alphavirus responsible for an unprecedented epidemic in countries of the Indian Ocean region, causing an acute and painful syndrome with strong fever, asthenia, skin rash, polyarthritis, and lethal cases of encephalitis. The most recent epidemic reemergences were documented in Kinshasa, (50,000 estimated cases in 1999–2000), in Indonesia (2001–2003), the Indian Ocean islands of Mayotte, Mauritius, Réunion, and the Seychelles (270,000 cases in 2005–2006 in La Réunion island), and in India (1.4 to 6.5 million estimated cases in 2006–2007). There is a critical lack of knowledge on the biology of CHIKV. In particular, virtually nothing is known about the interaction of CHIKV (and of most alpahaviruses) with human primary cells. We have studied the replication characteristics and the tropism of clinical CHIKV strains from La Réunion. We designed various assays and reagents to follow viral replication, and we report here that adherent cells (epithelial and endothelial cells, primary fibroblasts), as well as macrophages, are sensitive to infection. In contrast, blood cells did not allow viral replication. We also characterized viral entry pathways and sensitivity to interferons. These results provide a general insight into the interaction between CHIKV and its mammalian host. This paper is the result of a collaborative effort between numerous teams from Institut Pasteur, the Groupe Hospitalier Sud Réunion, and other institutions. Our aim was to establish a task force with multiple and complementary expertise on virology, immunology, and cell biology in order to characterize this enigmatic virus.

## Introduction

Chikungunya virus (CHIKV), an alphavirus belonging to the Togaviridae family, was first isolated from a febrile individual in Tanzania in 1952 [1,2]. CHIKV is transmitted to humans by several species of mosquitoes, with Aedes aegypti and A. albopictus being the two main vectors. The symptoms generally start 4–7 d after the bite. Acute infection lasts 1–10 d and is characterized by a painful polyarthralgia, high fever, asthenia, headache, vomiting, rash, and myalgia [2–4]. In Swahili, the term “chikungunya” means “the bent walker”. Indeed, in numerous patients, a chronic and incapacitating arthralgia persists for months. During the last 50 years, CHIKV has caused a number of outbreaks in East and South Africa and in Southeast Asia [5]. The most recent epidemic reemergences were documented in Kinshasa (50,000 estimated cases in 1999–2000) [6], Indonesia (2001–2003) [7], the Indian Ocean islands of Mayotte, Mauritius, Réunion, and the Seychelles (270,000 cases in 2005–2006 in La Réunion island) [5]), and India (1.4 to 6.5 million estimated cases in 2006–2007) [8–10].

The magnitude of the recent CHIKV intrusion in La Réunion island was unexpected, with 40% of the 785,000 inhabitants being infected in 2005–2006. This was the first CHIKV epidemic in a country with an occidental health care environment. Severe forms of the disease were reported, with about 250 fatal cases (corresponding to one death per 1,000 infections) [11]. Clinical cases, rarely or never described before, include lymphopenia, severe dermatological lesions, lethal hepatitis and encephalitis in adults (often elderly persons) and newborns, and fetal transmission during pregnancy leading to neonatal encephalopathy or abortion [4,12]. Many factors may explain why this virus recently spread so efficiently. CHIKV probably reached La Réunion for the first time, encountering a nonimmune and sensitive population. The mosquitoes involved in the local transmission *(A. albopictus)* are abundant and likely present a high vectorial capacity [13]. The circulating CHIKV strains might have also acquired particular replication properties. An extensive genome analysis of recent clinical isolates from the Indian Ocean outbreak identified unique molecular features when compared to the few previously available sequences from laboratory-adapted viruses [5]. In particular, changes in the viral envelope glycoprotein E1 were observed, potentially affecting viral fusion, assembly, and/or tropism.

There is a critical lack of knowledge on the biology of CHIKV, contrasting with related model alphaviruses like Sindbis virus (SINV), Semliki Forest virus (SFV), and Ross River virus (RRV); this probably reflects the fact that CHIKV has mostly afflicted persons in developing countries. Alphaviruses are enveloped, single-stranded, positive polarity RNA viruses [2]. Alphaviruses attach to poorly characterized receptors on many different cell types in various species. The E1 spike protein drives the fusion process, and E2 interacts with cellular receptors [14,15]. Individual viruses have different, but wide tropism, accounting somewhat for different disease patterns [2]. Viral entry generally occurs through receptor-mediated endocytosis, and fusion is dependent of endosomal acidification [16–19]. In the 1960s, CHIKV was used to stimulate type I interferon (IFN) production from chick embryo–like fibroblasts [20,21], and IFN most likely plays a prominent role in viral clearance and disease pathogenesis. Prior studies from the 1960s to 1980s have demonstrated that CHIKV replicates in various nonhuman cell lines, including Vero cells, chick embryo fibroblast–like cells, BHK21, L929, and Hep-2 cells [22–25], inducing generally a significant cytopathic effect (CPE) [2]. To our knowledge, the interaction of CHIKV with human primary cells has not been extensively characterized. Here, we report on the replication characteristics and the tropism of clinical CHIKV strains from La Réunion island and provide a general insight into the interaction between CHIKV and its human host.

## Results

### Analysis of CHIKV Replication

Virtually nothing is known about the interactions of CHIKV with human cells. We thus designed various assays to study CHIKV replication in cell cultures. Four clinical strains (isolated from patients from La Réunion), for which the genome has been sequenced [5], were analyzed. CHIKV-21 and CHIKV-27 strains were isolated, respectively, from the serum and cerebrospinal fluid of two newborns suffering from encephalopathy, whereas CHIKV-49 and CHIKV-115 came from two young adults with a classical form of the disease [5]. The four strains were genetically close, with only one polymorphic residue (at codon 226) in the E1 glycoprotein [5]. These Réunion isolates showed 1%–2% amino acid changes in their nonstructural and structural proteins when compared to the reference strain (isolated in 1952 in Tanzania). To avoid selection of minor laboratory-adapted variants [26], viruses were amplified only twice, in C6/36 mosquito cells, before characterization. Viral titers of C6/36 supernatants were measured using a standard procedure (infection of Vero cells with serial dilutions of supernatants to be tested, and measure of the appearance of a CPE) [27] and were confirmed with a focus immunoassay (see Materials and Methods). Titers reached 10^6^–10^7^ tissue culture infectious dose 50 (TCID50)/ml in C6/36 supernatants for the four strains.

**Figure 1:**
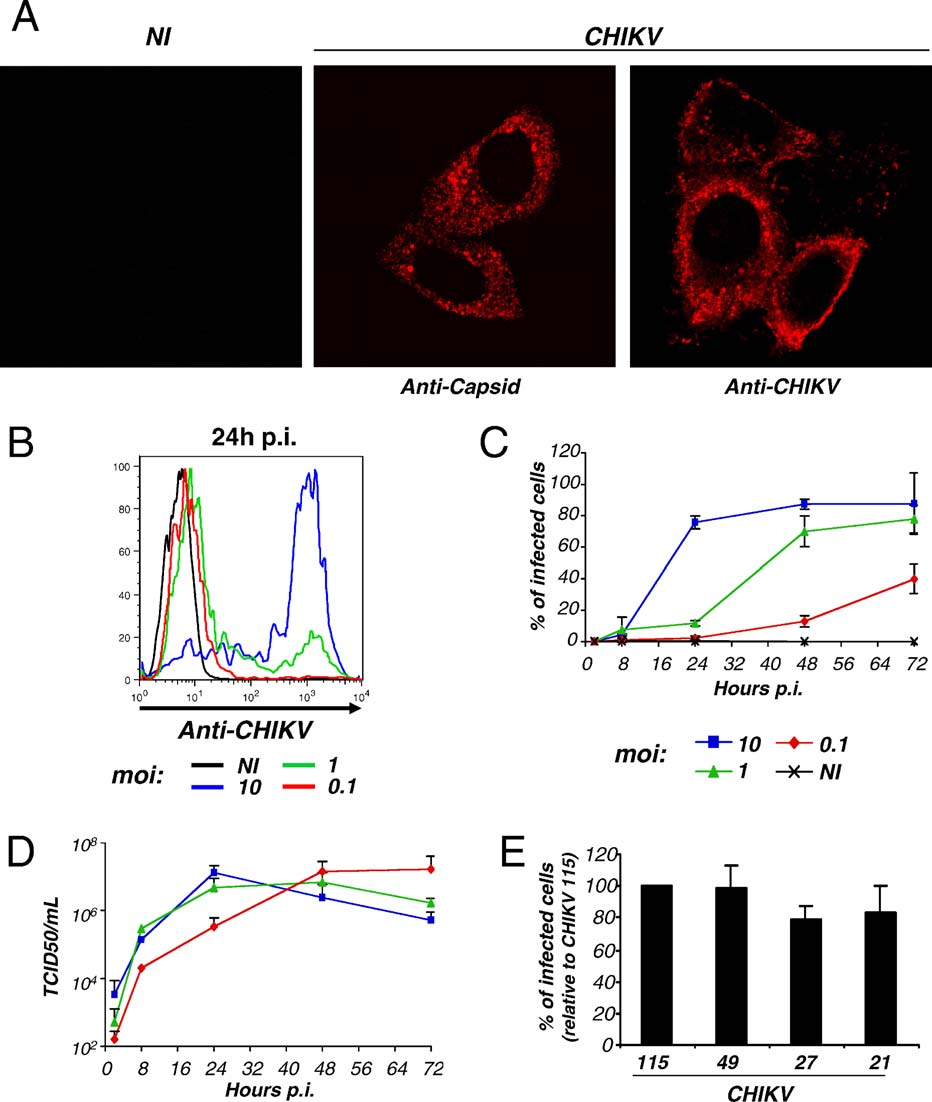
CHIKV Infection of HeLa Cells (A) HeLa cells were exposed to CHIKV-115. At 24 h pi, cells were fixed and stained with an anti-alphavirus capsid mAb or with polyclonal anti-CHIKV antibodies, and analyzed by confocal microscopy. (B) Quantification of CHIKV-infected cells by flow cytometry. HeLa cells were exposed to CHIKV-115 at the indicated moi. At 24 h pi, cells were fixed, permeabilized, stained with anti-CHIKV antibodies, and analyzed by flow cytometry. Similar results were obtained with the anti-capsid mAb. (C) Kinetic analysis. HeLa cells were infected as described in (B) and analyzed at the indicated time points. Data are a compilation (with standard deviation [SD]) of three independent experiments. (D) Viral release in supernatants*.* Levels of infectious virions in supernatants were measured by limiting dilution on Vero cells and are expressed as TCID50/ml. Data are a compilation (with SD) of three independent experiments. (E) Comparison of the infectivity of four CHIKV strains. Mean ±SD of five independent experiments are depicted, with 100% corresponding to values obtained with CHIKV-115, at days 1 and 2 pi, with mois of 10 and 1. NI, noninfected cells.

We first used the HeLa epithelial cell line as target. HeLa cells were exposed to CHIKV-115 at a high multiplicity of infection (moi), and the presence of viral proteins was examined by immunofluorescence, after staining with a monoclonal antibody directed against a conserved epitope of alphavirus capsid (C) [28]. At 2 h post-infection (pi), a weak punctuate staining was observed, likely corresponding to incoming virions bound or internalized by target cells (not shown). At 24 h pi, the anti-C staining was more intense throughout the cytosol (Figure 1A). Mouse polyclonal anti-CHIKV antibodies also gave a strong signal in infected cells, staining both the cytosol and plasma membrane (Figure 1A). Quantification of the fraction of infected cells was then performed by flow cytometry. At a high viral dose (moi 10), 80%–100% of the cells were positive for CHIKV antigens at 24–48 h pi (Figure 1B and 1C). Productive infection and expression of viral proteins increased according to the initial inoculum (Figure 1B). A kinetic analysis showed that infection spread rapidly in cultures, thus indicating that HeLa cells were capable of producing infectious virus (Figure 1C). At high mois (10 and 1), about 50% of the cells were infected as soon as 8–16 h pi, whereas there was a delay in the appearance of CHIKV-positive cells at a lower moi (0.1) (Figure 1C). This rapid viral propagation was associated with release of high levels infectious virus in supernatants, reaching 10^6^–10^7^ TCID50/ml at 24–48 h pi for the high mois (10 and 1) and at 48–72 h pi for the low moi (0.1) (Figure 1D). Similar results were obtained with the four CHIKV strains, indicating that they behave similarly in HeLa cells (Figure 1E).

**Figure 2:**
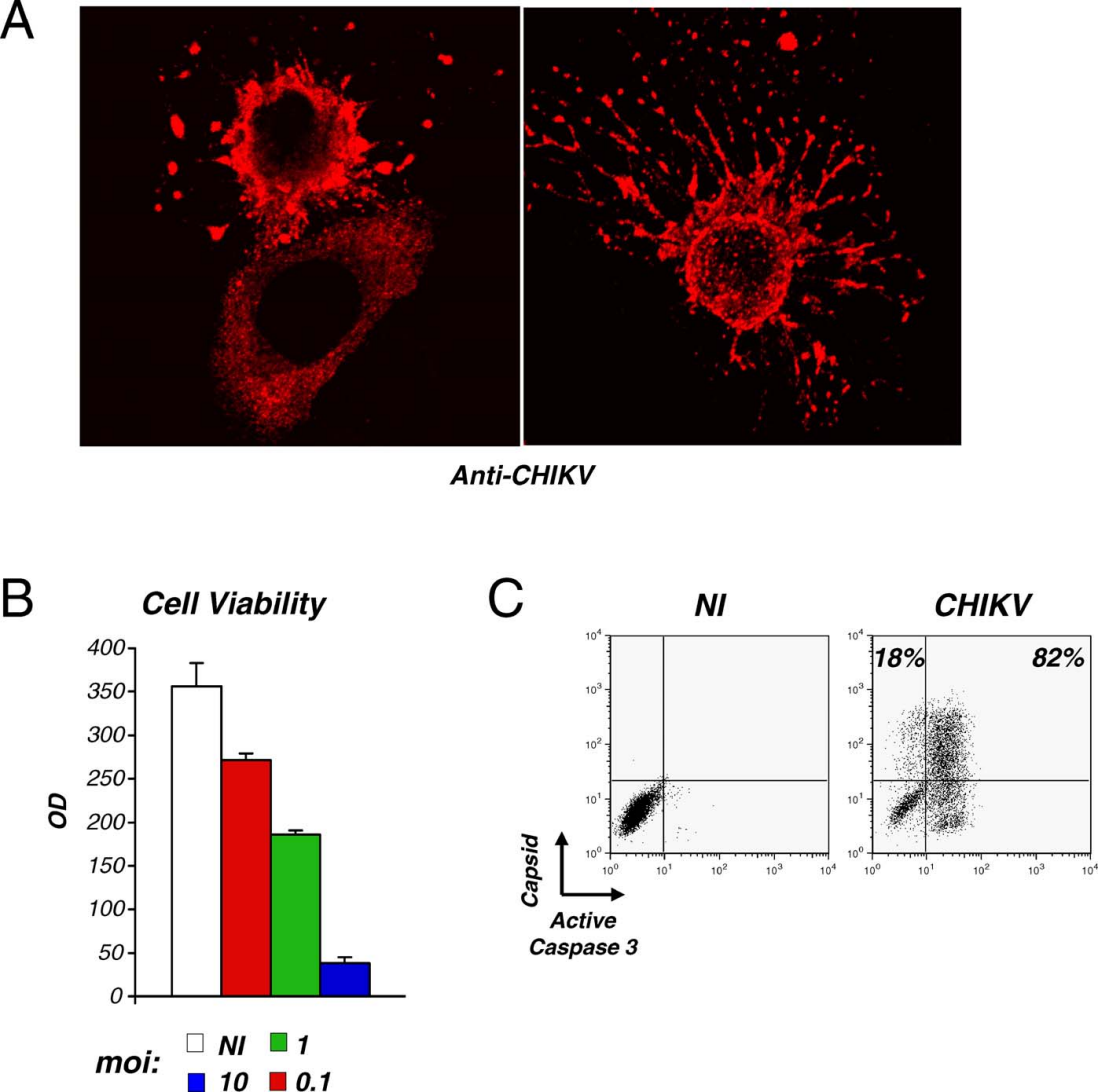
CHIKV Is a Cytopathic Virus (A) CHIKV-infected dying cells. At 24 h after CHIKV infection, HeLa cells were fixed, stained with anti-CHIKV antibodies, and analyzed by confocal microscopy. (B) Viability of cell cultures. HeLa cells were infected with CHIKV at the indicated moi. After 24 h pi, cell viability was measured in a colorimetric assay (MTT cell viability test). Data are mean ±SD of triplicates and representative of five independent experiments; (C) Apoptosis of CHIKV-infected cells. At 24 h pi (moi 10), HeLa cells were doubly stained with anti-active caspase-3 and anti-capsid antibodies and analyzed by flow cytometry. The percentage of caspase-3 positive and negative cells among CHIKV positive cells is depicted. Similar results were obtained with another marker of apoptosis (TUNEL, not shown). Data are representative of three independent experiments. NI, noninfected cells.

Alphaviruses are known to produce a marked CPE in vertebrate cell cultures [2]. This was also the case for CHIKV in HeLa cells, in which extensive cell death occurred at 24 h pi (at the high moi), as observed by immunofluorescence and measured in a colorimetric assay (MTT cell viability test) (Figure 2A and 2B). Again, this CPE varied depending on the viral inoculum (Figure 2B). Dying cells were positive for CHIKV antigens, and a large amount of cell debris was visible (Figure 2A). Death of infected cells was associated with apoptosis, as assessed by the presence of numerous active caspase-3 and CHIKV double-positive cells (Figure 2C). In three independent experiments, 75% ±5% of CHIKV-positive cells also expressed an active caspase-3, at day 1 pi. Infected cells were also positive for another marker of apoptosis (TUNEL staining, not shown).

Of note, we repeatedly observed an apparent discrepancy between the number of productively infected cells analyzed by flow cytometry, and virus titers in supernatants (compare, for example, Figure 1C and 1D when a low moi is used). This is likely the consequence of a rapid and extensive mortality of infected cell populations, especially at high mois: the percentage of infected cells is actually measured on living cells. It is also possible that the flow cytometry assay mostly detects cells expressing high levels of viral proteins.

**Figure 3:**
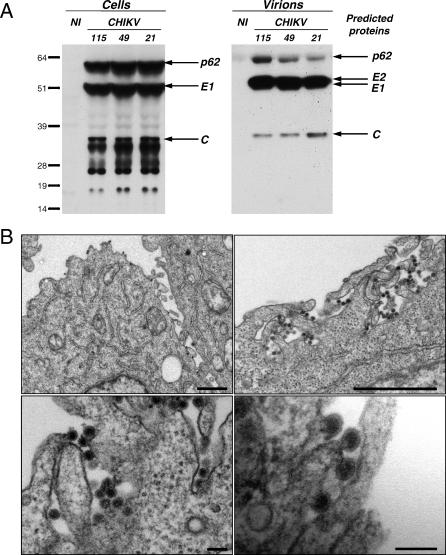
Assembly and Release of CHIKV (A) Western blot analysis of CHIKV proteins. HeLa cells were exposed to the indicated CHIKV strains (moi 10). Cell lysates and pelleted supernatants were analyzed by Western blot 24 h pi, with a mix of anti-capsid mAb and anti-CHIKV antibodies. The predicted viral proteins are indicated on the right. (B) Gallery of electron micrographs of CHIKV-infected HeLa cells. Cells were analyzed at 36 h pi. CHIKV mostly buds at the plasma membrane of HeLa cells. Viral particles were not detected in noninfected cells (not shown). Bars represent 1 μm in the upper panels and 100 nm in the lower panels. NI, noninfected cells.

We also characterized CHIKV by Western blot analysis. Various viral proteins were detected in lysates of CHIKV-infected HeLa cells and in viral particles pelleted from the supernatants (Figure 3A). From their apparent molecular weight, major viral proteins in cell lysates were likely the p62 precursor of the envelope glycoprotein E2 (62 kDa), the envelope glycoprotein E1 (52 kDa), and the capsid C (36 kDa) [25]. Other bands in cell lysates probably corresponded to nonstructural and structural proteins, as well as precursors or intermediate cleavage products [2,25,29]. The three main bands in pelleted supernatants likely corresponded to low levels of p62, the E2–E1 doublet (56 and 52 kDa) and C (Figure 3A). A similar Western blot profile was obtained with sera from patients (not shown), indicating that these viral proteins are synthesized and immunogenic in infected individuals.

An electron microscope study revealed the presence of numerous viral-like particles in CHIKV-infected cells (at 36 h pi) (Figure 3B). Virions displayed the characteristic features of alphaviruses [30,31]: a size of 50–70 nm, an icosahedral-like shell of C proteins, and the presence of viral envelope glycoproteins on their surface (Figure 3B). Although the intracellular site of alphavirus budding has been debated [30,31], our analysis indicates that CHIKV appears to bud mostly at the plasma membrane of HeLa cells.

Altogether, these results demonstrate that CHIKV efficiently propagates in HeLa cells, causing a rapid death of infected cells by apoptosis. Viral replication can be evaluated with various methods, including detection of infected cells by immunofluorescence or flow cytometry, measurement of viral release in supernatants, and quantification of cell viability.

### Cell Tropism of CHIKV

**Figure 4:**
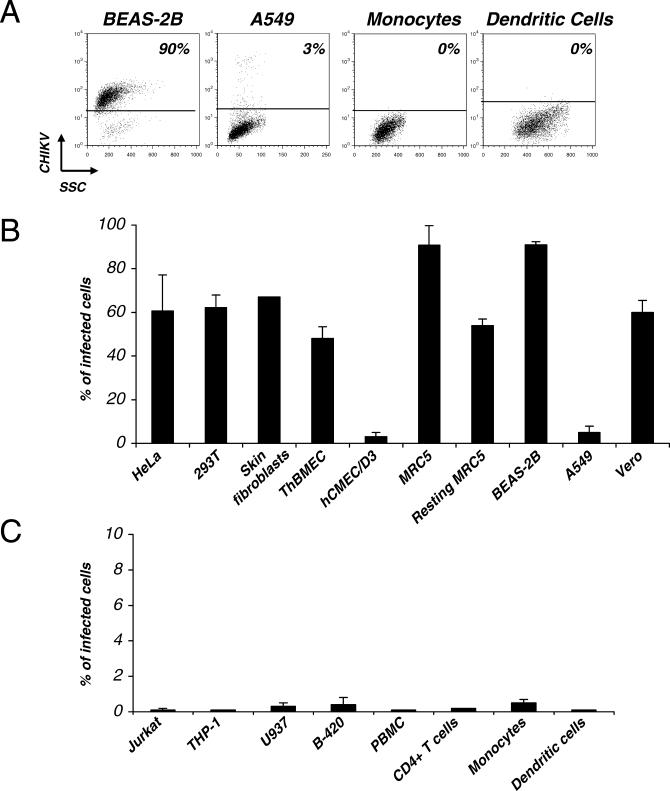
Cell Tropism of CHIKV (A–C) The indicated human cell lines or primary cells were exposed to CHIKV at an moi of 10. At 24 h pi, cells were fixed, stained with anti-CHIKV antibodies, and analyzed by flow cytometry. The percentage of CHIKV-infected cells is indicated. Data are representative of at least three independent experiments. For primary cells, at least three different donors were analyzed. (A) Examples of sensitive and refractory cell types. (B) Sensitivity of adherent cells to CHIKV infection. (C) Sensitivity of primary blood-derived cells to CHIKV infection. The indicated cell lines, as well as nonactivated PBMCs, activated CD4+ lymphocytes, monocytes, and monocyte-derived DCs were analyzed.

We tested a panel of immortalized and primary human cells for their ability to replicate CHIKV. The epithelial-derived cell lines HeLa, 293T, and BEAS-2B, as well as primary fibroblasts (Hs 789.Sk skin cells and MRC5 lung cells), were highly susceptible to the virus. After 24 h of infection, more than 60% of the cells were CHIKV-positive by flow cytometry (Figure 4A and 4B) and by immunofluorescence analysis (not shown). CHIKV infection was associated with extensive cell mortality and with the release of high levels of infectious virus in supernatants (not shown). Interestingly, resting MRC5 cells, obtained by maintaining confluent cultures in serum-free medium for at least 10 d before viral exposure (95% of the cells at G0/G1 by flow cytometry analysis of the cell cycle, not shown), were also susceptible to infection (Figure 4B). Therefore, CHIKV replicates in dividing and nondividing cells. We also identified an epithelial cell line (A549 alveolar cells), which was resistant to CHIKV infection. Less than 5% of the cells express CHIKV antigens at day 2 pi, without any CPE (Figure 4A and 4B). We then tested two human endothelial cell lines, TrHBMEC and hCMEC/D3, isolated from the bone marrow and the brain, respectively [32,33]. Interestingly, TrHBMEC cells were readily infected and killed by CHIKV, whereas hCMEC/D3 cells were much more resistant (Figure 4B). With hCMEC/D3, only 1% of the cells were CHIKV+ at 24 h pi, this fraction did not increase over time, and no obvious CPE was observed (not shown). Of note, monkey Vero cells were also sensitive to CHIKV infection (Figure 4B).

The acute phase of chikungunya disease is associated with a high peak of viremia, which can reach 10^8^ viral RNA copies/ml (P. Laurent, K. Le Roux, P. Grivard, G. Bertil, F. Naze, et al., unpublished data). We thus asked whether blood-derived cell lines support CHIKV replication. Jurkat (CD4+ T lymphoid cells), THP-1 and U937 (monocytoid cells), and B-420 (EBV-transformed B cells) were refractory to CHIKV infection (Figure 4B). We then examined the sensitivity of primary blood cells. We tested nonactivated and PHA-activated peripheral blood mononuclear cells (PBMCs), as well as purified cell subsets, including CD14+ monocytes, activated CD4+ T cells, immature and mature monocyte-derived dendritic cells (DCs). None of these cell types were sensitive to CHIKV infection, and viral exposure did not induce any apparent toxicity in these cells (Figure 4C).

**Figure 5:**
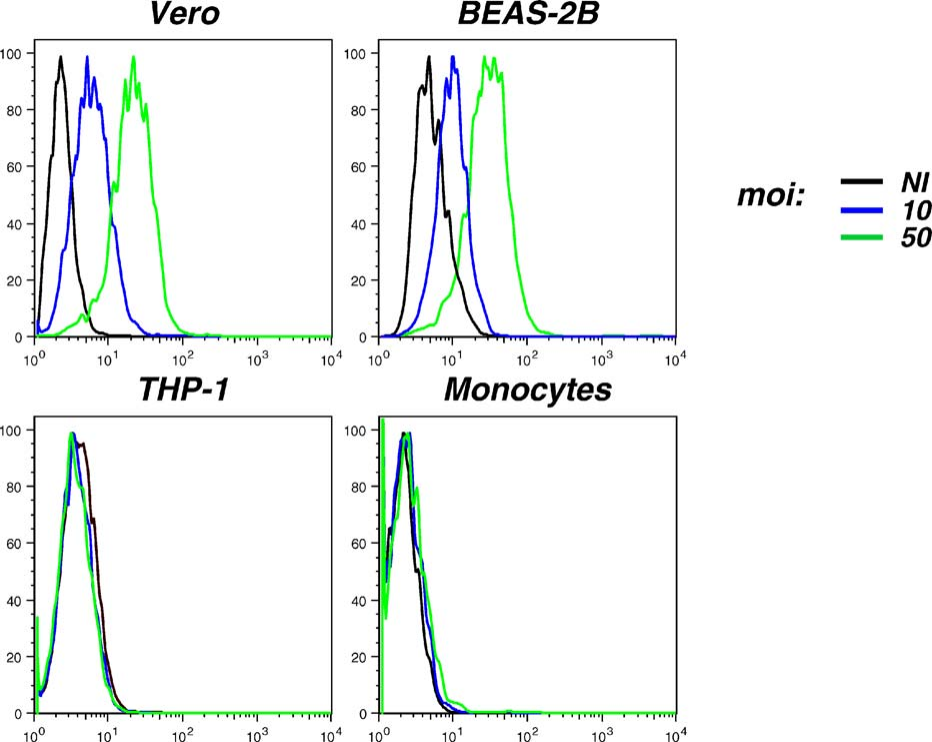
Binding of CHIKV to Target Cells The indicated human cell lines or primary cells were exposed to CHIKV at the indicated moi for 1 h at 4 °C. Cells were fixed, stained with anti-CHIKV antibodies, and analyzed by flow cytometry. Data are representative of three independent experiments. NI, noninfected cells.

We documented further the tropism of CHIKV by designing an assay to characterize viral binding to these various sensitive and restrictive cells. Target cells were incubated at 4° C for 1 h with CHIKV (at mois of 10 and 50), and the amount of bound viral materials was measured by flow cytometry after staining with anti-CHIKV antibodies. Viral binding varied depending on the cell type. An example of two cell types with strong binding (Vero and BEAS-2B cells) and two others with undetectable binding (THP-1 and primary monocytes) is provided in Figure 5, while Table 1 summarizes results obtained with the whole panel of tested cells. Interestingly, there was generally a good correlation between the efficiency of viral binding and sensitivity to infection (Table 1).

**Table 1:**
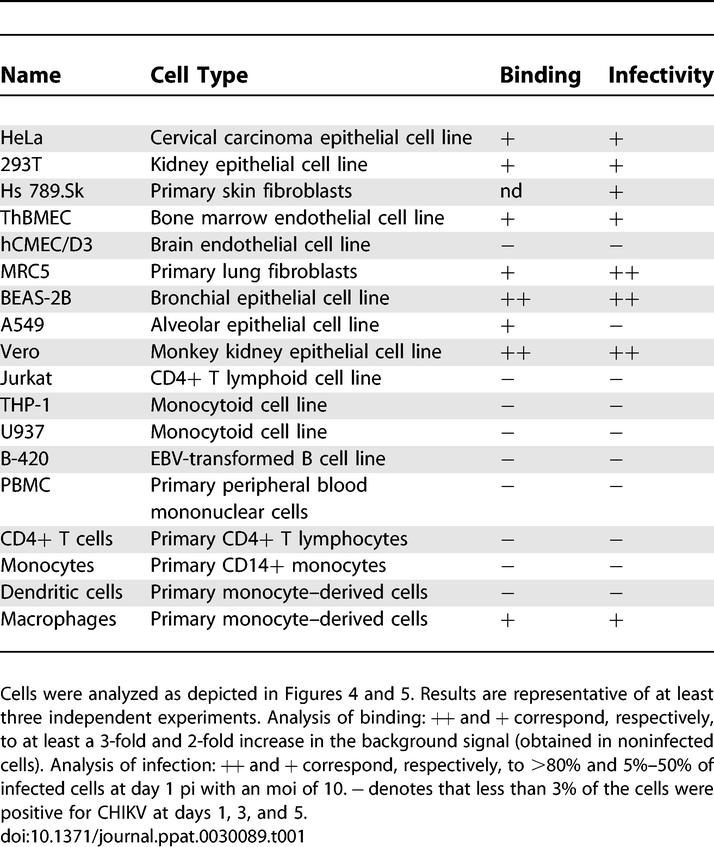
Analysis of CHIKV Binding and Sensitivity to Infection of a Panel of Adherent and Nonadherent Cells

In conclusion, these results indicate that CHIKV readily infects most (but not all) of the adherent cell lines or primary cells tested, whereas blood cells are refractory to the virus. The absence of infection is generally due to a poor binding of incoming CHIKV to target cells. Of note, similar results were obtained with the four CHIKV strains analyzed and with viruses produced in either mosquito (C6/36) or mammalian (HeLa) cells, indicating that there are no obvious differences in the tropism of these isolates in cell culture.

### CHIKV Productively Infects Human Primary Macrophages

**Figure 6:**
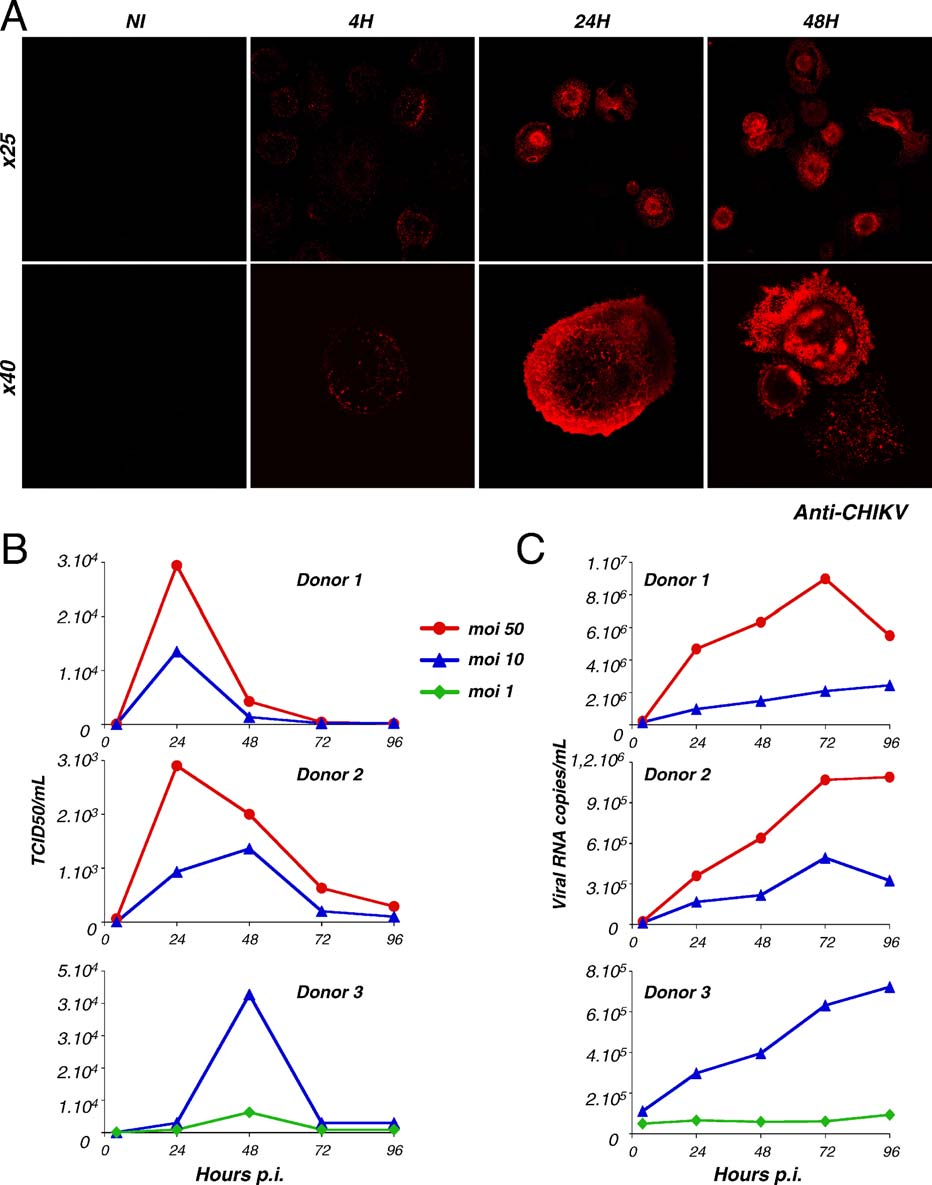
CHIKV Productively Infects Human Primary Macrophages (A–C) Human monocyte–derived macrophages were exposed to CHIKV for 4 h and extensively washed, and CHIKV replication was analyzed by different methods. Data are representative of at least four independent experiments, with cells from eight different donors. (A) CHIKV-infected macrophages. Cells were infected with CHIKV at an moi of 10. At the indicated time points, cells were stained with anti-CHIKV antibodies and analyzed by confocal microscopy. Two magnifications are depicted (objectives ×25 and ×40). NI, noninfected cells. (B) Release of infectious virus in supernatants*.* Macrophages were infected at various mois as stated. At the indicated time points, levels of infectious virions in supernatants were measured by limiting dilution on Vero cells. Results are expressed as TCID50/ml. Macrophages from three representative donors are depicted. (C) Viral RNA in supernatants*.* Levels of viral RNA in supernatants from the same experiment depicted in (B) were measured by real-time PCR.

Since monocytes are insensitive to CHIKV, we asked whether macrophages allow CHIKV replication. Monocyte-derived macrophages were prepared from PBMCs of healthy donors [34]. Cells expressed CD14, CD4, and CCR5 (markers of the macrophage lineage), strongly adhered to bottom wells, and displayed high phagocytic activity (not shown). Macrophages were exposed to CHIKV for 2–4 h at 37 °C, the unbound virus was removed by extensive washing, and viral replication was assessed over time by immunofluorescence analysis and by measuring the release of infectious virus (Figure 6). At 4 h pi, some faint intracellular dots were visible, likely corresponding to incoming virions. At a later time, (24 h pi), productively infected cells, with a strong CHIKV signal, were detected in the cultures. Depending on the donors, approximately 5%–50% of the cells were positive, with either the anti-C monoclonal antibody (mAb) (not shown) or polyclonal anti-CHIKV antibodies, at 24–48 h pi (Figure 6A). Infectious viral material was released in the supernatants, reaching 10^4^–10^5^ TCID50/ml at 24–48 h, and then slowly declining over time (Figure 6B). Similar results were obtained with cells from eight different donors and with various CHIKV strains (not shown). We also quantified viral RNA release over time with a real-time PCR assay (P. Laurent, K. Le Roux, P. Grivard, G. Bertil, F. Naze, et al., unpublished data). A level of 10^6^–10^7^ viral RNA copies was reached at 72 h pi in macrophages from three different donors. Results with cells from two donors, infected at mois of 10 and 50, and from one donor infected at mois of 1 and 10, are depicted Figure 6C. Interestingly, the amount of infectious virions peaked at day 1 or 2 and then decreased, whereas the production of viral RNA did not decline so rapidly. This suggests that infected, dying macrophages may release noninfectious viral RNA. Altogether, these results indicate that CHIKV productively infects monocyte-derived macrophages. The extent of viral replication in macrophages is, however, lower than in HeLa cells.

### CHIKV Entry Pathways

**Figure 7:**
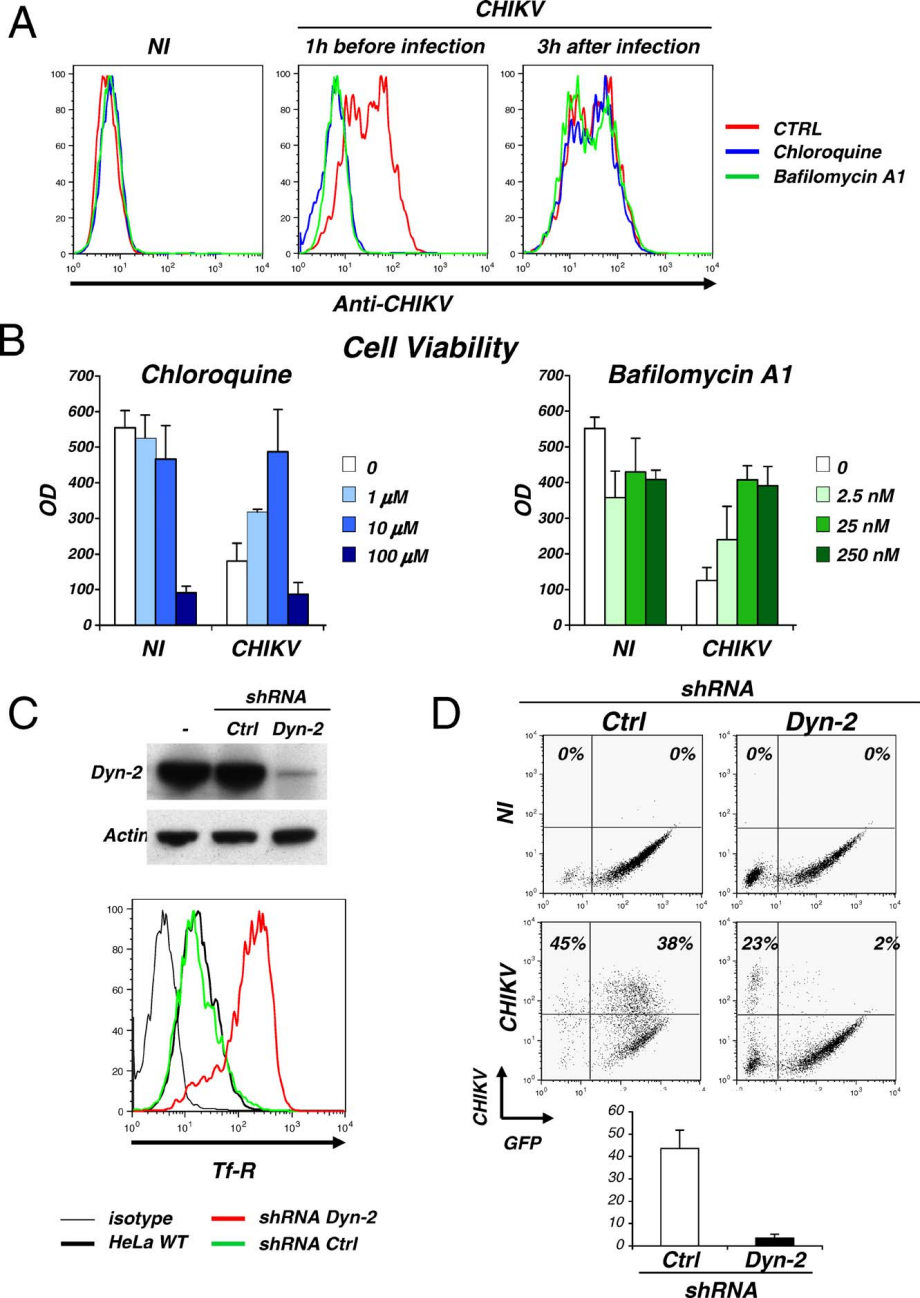
CHIKV Entry Pathway (A and B) CHIKV infection requires a low endosomal pH for entry. HeLa cells were pretreated 1 h before infection with chloroquine (10 μM) or bafilomycin-A1 (25 nM) and exposed to CHIKV (moi 10), or treated with the two drugs 3 h after viral exposure. (A) Inhibition of CHIKV replication by chloroquine and bafilomycin-A1. At 24 h pi, HeLa cells were stained with mouse anti-CHIKV antibodies and analyzed by flow cytometry. Ctrl, control. (B) Inhibition of CHIKV CPE by chloroquine and bafilomycin-A1. Cell viability was measured in a colorimetric assay (MTT cell viability test) 24 h pi. (C) Downregulation of Dyn-2 expression by shRNAs. HeLa cells were transduced with a lentiviral vector expressing GFP and a shRNA against Dyn-2 or an unrelated protein as a control (Ctrl). Upper panel: The Western blot shows the downregulation of Dyn-2 expression. Lower panel: surface levels of transferrin receptor (Tf-R) were upregulated in the absence of Dyn-2. Flow cytometry analysis was gated on transduced GFP+ cells. (D) Dyn-2 is required for CHIKV replication. HeLa cells lacking Dyn-2 are resistant to CHIKV. Infected HeLa cells (moi 10, 24 h pi) were stained with anti-CHIKV antibodies and analyzed by flow cytometry. The percentage of CHIKV+ cells among GFP+ and GFP− cells is depicted. One representative experiment out of three is shown in the upper panel. A mean ±SD of four independent experiments is shown in the lower panel. NI, noninfected cells.

Alphaviruses like SINV and SFV penetrate target cells through clathrin-mediated endocytosis, the low pH of the endosomal compartment promoting conformational changes of envelope glycoprotein and viral fusion [15,16,18]. We examined whether CHIKV infection requires a low endosomal pH for entry. HeLa cells were treated with two compounds that impair intracellular vesicle acidification, bafilomycin-A1, an inhibitor of vacuolar proton-ATPases [35], or the weak base chloroquine. Both compounds potently inhibited the appearance of CHIKV-positive cells and CHIKV-associated CPE (Figure 7A and 7B). Bafilomycin-A1 was efficient at doses from 2.5 to 250 nM, without obvious cytotoxicity, whereas the active concentration range of chloroquine was much narrower: this compound fully inhibited CHIKV infection at 10 μM, but was toxic at 100 μM (Figure 7B). Of note, no inhibitory effect was observed if addition of these two compounds was delayed until 3 h after CHIKV exposure (Figure 7A). This suggests that the requirement for an acidic compartment is a feature of an early step of the viral cycle.

We studied further the role of the clathrin endocytic pathway by knocking down the expression of dynamin-2 (Dyn-2), a key protein required for the formation of clathrin-coated pits and vesicles, and of caveolae [18,36,37]. This was achieved by using lentiviral vectors expressing EGFP and a short hairpin RNA (shRNA) against Dyn-2 [38]. Transduction of HeLa cells inhibited Dyn-2 expression in EGFP+ cells (Figure 7C), with the expected functional implications, as evidenced by a significant increase in transferrin receptor surface expression (Figure 7C). Interestingly, HeLa cells lacking Dyn-2 were largely refractory to CHIKV-infection, whereas cells transduced with a vector coding for a control shRNA were normally infected (Figure 7D). In four independent experiments, the absence of Dyn-2 was associated with a 20-fold reduction in the sensitivity of HeLa cells to CHIKV (Figure 7D). Altogether, these experiments strongly suggest that CHIKV requires an active clathrin-dependent pathway of internalization and targeting of a low pH endosomal compartment for successful infection.

### CHIKV Replication Is Sensitive to IFNs

IFN was first discovered in 1957 as an undefined substance with antiviral activity. Work within the last several decades has defined two classes of antiviral substances: type I IFNs (IFNα/β), which signal via a shared receptor, and type II IFN (IFNγ), which signals via a unique IFNγ receptor. Interestingly, in the 1960s, CHIKV was used to stimulate IFN production from chick embryo fibroblast–like cells [20,21], but the effect of IFNs on CHIKV infection has not been assessed. We determined the potential role of IFNs in limiting CHIKV infection by using the HeLa infection system. Cells were pretreated with either IFNα_1b_, IFNβ_1a_, or IFNγ for 6 h, at a dose ranging from 10 to 1,000 IU/ml, and viral replication was then analyzed by flow cytometry. Interestingly, the three IFNs potently inhibited the expression of viral proteins (Figure 7A). Moreover, treatment with IFN strongly decreased virus-induced CPE (Figure 7B). Similar results were obtained with the four viral strains, indicating that CHIKV is highly sensitive to the antiviral effect of IFNs. Of note, CHIKV replication was also strongly inhibited by IFN in macrophages (not shown), indicating that the antiviral effect of IFN is also visible in primary cells.

## Discussion

We describe here the replication features of CHIKV, a virus responsible for recent epidemic outbreaks in India and other countries in the Indian Ocean region. So far, this virus species has not been scrutinized using modern-day techniques. We have designed a panel of assays in order to follow viral replication and to describe the cellular tropism of CHIKV in culture experiments. We report that CHIKV replicates in various human adherent cells, including epithelial and endothelial cells, and primary fibroblasts and macrophages. In contrast, T and B lymphocytes and monocyte-derived DCs are not susceptible. Viral entry occurs through a pH-dependent, endocytic pathway. The life cycle of this virus is short: as soon as 8–16 h pi, numerous newly infected cells can be detected, which release high levels of progeny virions. Viral titers in supernatants reach 10^5^–10^8^ TCID50/ml, depending on the cell type. CHIKV is highly cytopathic for mammalian cells, inducing apoptosis of infected cells. Moreover, CHIKV replication is significantly inhibited by type I and II IFNs. These characteristics generally correspond to those of other alphaviruses, among which the most studied are SINV, SFV, and RRV [14,31,39,40]. The alphavirus genus contains over 20 members, which likely diverged 2,000 to 3,000 years ago [40]. Some alphaviruses are nonpathogenic in humans, whereas others cause different diseases with various intensities, and can be broadly divided into the American encephalitis viruses and the globally distributed arthritogenic viruses [2,41]. Each virus type has thus likely evolved its own way of interacting with the host.

**Figure 8:**
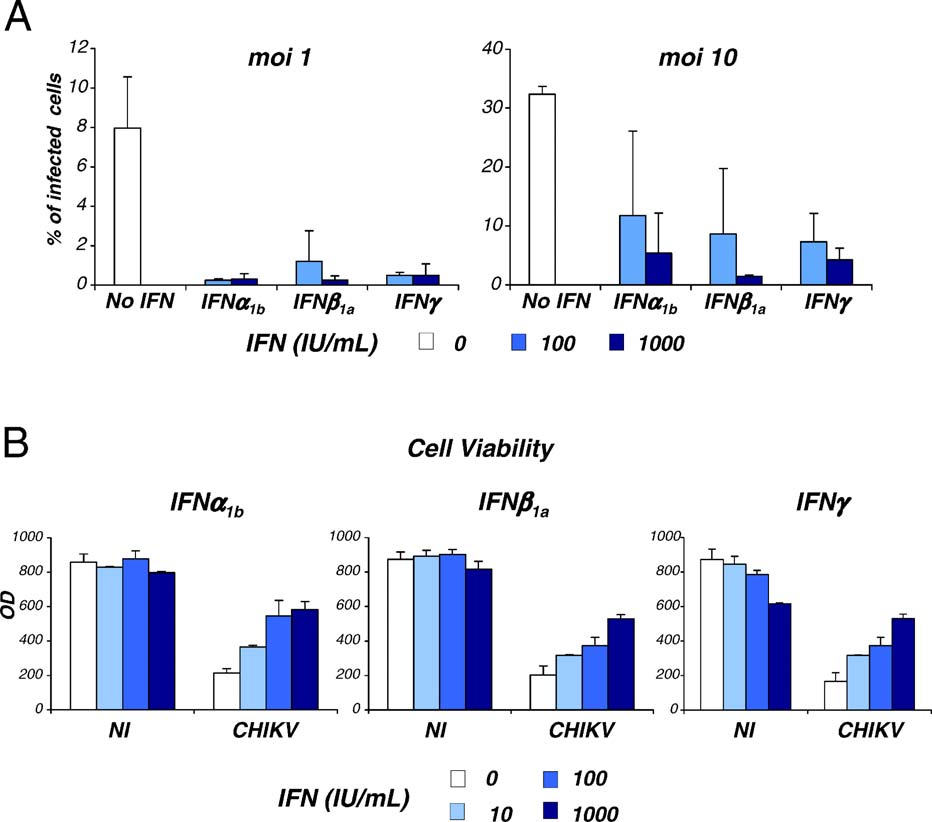
IFNs Inhibit CHIKV Infection HeLa cells were pretreated 6 h before infection with the indicated doses (IU/mL) of IFN. IFNα_1b_ , IFNβ_1a_, and IFNγ were tested. Cells were then exposed to CHIKV at two mois (10 and 1). (A) Inhibition of CHIKV replication. At 24 h pi, HeLa cells were stained with anti-CHIKV antibodies and analyzed by flow cytometry. (B) Inhibition of CHIKV CPE. Cell viability was measured in a colorimetric assay (MTT cell viability test) 24 h pi (moi of 10). Data are representative of three independent experiments.

So far, alphaviruses have been mostly studied in murine and other animal cells. In particular, the interaction of CHIKV with human cells has not been characterized. We have reported here that some human epithelial and endothelial cells and fibroblasts are sensitive to CHIKV. Together with the recent finding that CHIKV replicates in human muscle satellite cells, and not in differentiated myotubes [42], our results indicate that CHIKV displays a rather wide tropism for adherent cells. However, CHIKV neither efficiently replicated in hCMEC/D3 endothelial cells nor in A549 epithelial cells. The identification of such CHIKV-resistant cells will be useful for further studies aimed at deciphering entry or post-entry viral events. For instance, the cellular receptors for CHIKV are not known. We generally observed a direct correlation between viral binding and infection of target cells (Table 1). Our results suggest that hCMEC/D3 cells are not infected, because of the absence of surface binding receptors. The situation may be different with A549 cells, which are refractory to infection but still able to bind viral particles. A549 cells may either nonspecifically bind incoming virions (through receptors not involved in productive entry), or restrict infection at a post-binding step.

As for other alphaviruses [14,16,43], CHIKV entry is pH-dependent. Viral replication is blocked by compounds inhibiting endosomal acidification and likely requires the clathrin machinery, as demonstrated by the requirement of Dyn-2 for viral replication. Dyn-2 also controls clathrin-independent uptake via caveolae [38,44], and it will be thus of interest to examine whether caveolae are also involved in CHIKV entry. Although chloroquine blocked CHIKV replication, the therapeutic (antiviral) index of chloroquine in cell cultures is rather narrow (Figure 7); thus, one should be cautious when proposing the use of chloroquine as an antiviral treatment in infected individuals.

CHIKV is highly cytopathic in human cell cultures, and infected cells rapidly undergo apoptosis. Alphavirus replication strongly affects fundamental cell physiology processes, with an inhibition of cellular transcription and translation, and a redirection of cellular resources towards the synthesis of viral proteins and genomes [45]. Induction of apoptosis by SINV occurs at the level of cell entry, without requiring virus replication [46,47]. Determining whether this is also the case for CHIKV, and whether apoptosis of CHIKV-infected cells is involved in pathogenesis, will require further investigations.

IFNs are essential components of the innate immune system, protecting against alphaviral disease [14,48,49]. A 2005 study proposed a correlation between alphavirus virulence and resistance to type I IFNs, as reported for the Eastern equine encephalitis virus [49]. We show here that the situation may be different for CHIKV, since this pathogenic virus retains full sensitivity to type I and II IFNs in cell cultures. This strongly suggests that the innate immune system controls the virus and is responsible for the rapid decline (a few days) of viremia observed during the acute phase of infection. It will be useful to determine which IFN-induced proteins mediate the inhibition of CHIKV replication. An interesting candidate is ISG15, which was recently shown to function as a critical antiviral molecule against SINV in mouse [50].

In contrast to adherent cells, primary lymphocytes, T cell, B cell, and monocytoid lines did not allow CHIKV replication. Similar results have been described for RRV and SFV [51,52]. Retroviral vectors pseudotyped with envelope glycoproteins from these two alphaviruses efficiently transduced adherent cells, but failed to infect lymphocytes and monocytes [51,52]. This is probably due to the lack of adequate receptor expression in lymphocytes and monocytes. One can speculate that CHIKV receptors are also absent in these cells, as supported by the lack of virus binding in our assay. Since a high peak of viremia occurs during the acute phase of chikungunya disease (P. Laurent, K. Le Roux, P. Grivard, G. Bertil, F. Naze, et al., unpublished data), we sought to determine whether PBMCs from acutely infected individuals harbor CHIKV. We did not observe detectable levels of viral RNA in the blood cell fraction from three individuals with a plasmatic viral load ranging from 10^5^– 10^8^ RNA copies/ml (not shown). Therefore, PBMCs are not sensitive to CHIKV in vitro, and are probably not infected in vivo.

Human monocyte–derived DCs were not sensitive to CHIKV replication. Multiple parameters regulate the ability of alphaviruses to infect DCs; for instance, RRV envelope glycoproteins allow infection of murine, and not human DCs [52]. Infection of human DCs by a SINV vector is determined by a single amino acid substitution in E2 [53]. In a mouse model, the in vivo targeting of Venezuelan equine encephalitis virus to skin DCs is required for pathogenesis, and is also regulated by amino acids in E2 [54]. On the other hand, mosquito cell–derived RRV and Venezuelan equine encephalitis virus exhibit enhanced infection of murine myeloid DCs, compared to mammalian cell–derived preparations [55]. This is due to a better induction of type I IFN by viruses produced in mammalian cells [55]. In our hands, monocyte-derived DCs were insensitive to CHIKV produced in either mosquito (C6/36) or human (HeLa) cells. Similar results were observed with immature and mature DCs, and exposure of immature DCs to CHIKV (from C6/36 or HeLa cells) did not induce their maturation, nor promote type I IFN production (not shown). Our results strongly suggest that monocyte-derived DCs are intrinsically resistant to CHIKV. Further work should examine the sensitivity of other DC subsets, like plasmacytoid DCs and Langerhans cells.

We demonstrate here that in contrast to DCs, human primary macrophages are susceptible to CHIKV. Infection of macrophages is associated with release of infectious viral progeny in the supernatants. This process is less efficient than in HeLa cells, with only 5%–50% of the cell population being positive, and viral titers plateauing at 10^4^–10^5^ pfu/ml, depending on cell donors. This restricted replication may be due to the secretion of IFN or other cytokines by infected macrophages. RRV, another arthritogenic virus, also infects macrophages [41,56–58]. Macrophages have been implicated in the pathogenesis of RRV disease, at least in a mouse model of infection. In this model, infiltrates of inflammatory macrophages are observed in muscles and joints [41,59], and treatment of mice with macrophage-toxic agents abrogated symptoms [60]. Moreover, RRV can induce persistent productive infection of macrophages in cultures [56,61]. Antibody-dependent enhancement of RRV infection in monocyte/macrophage cell lines has been reported, but whether this process plays a role in vivo remains to be proven [41,56].

There are, however, noticeable differences between RRV and CHIKV interactions with macrophages. First, RRV has been mostly studied using murine monocytic/macrophage cells, or human monocytoid cells, and to our knowledge, there is no evidence for RRV infecting primary human macrophages. Moreover, RRV [56], and not CHIKV (Figure 4B), infects monocytoid cells such as U937 cells. CHIKV induces a sudden onset of severe arthritis and fever, whereas RRV-induced symptoms are generally mild and more gradual [40]. Whether these differences are linked to a differential interaction of the viruses with macrophages in vivo remains to be determined.

During the recent outbreak of CHIKV in La Réunion, an evolution of the viral genome was reported when comparing initial and later circulating strains. In particular, the selection of a mutant of the E1 glycoprotein (A226V) was noted [5]. In our studies, we have used four clinical CHIKV strains with different sequences. All the variants behave similarly in our assays, suggesting that the reported evolution is not due to the selection of viruses with a modified tropism for human cells.

Overall, our results provide fresh insights into the replication of alphaviruses and the pathogenesis of CHIKV. We identified a panel of human cell types sensitive to CHIKV, and our next aim is to further characterize how CHIKV interacts with these cells. It will be of interest to determine how DCs, macrophages, and other cell types sense CHIKV, and which cytokines are produced during the encounter. It will be also useful to correlate our results with the situation in vivo, for example, by determining in biological samples from CHIK-infected individuals which cells harbor the virus during both acute and chronic phases of the disease.

## Materials and Methods

### Cells and reagents.

HeLa cells, 293T cells, primary skin cells (Hs 789.Sk, American Type Culture Collection [ATCC] number 7518; http://www.atcc.org), TrHBMEC, BEAS-2B cells, MRC5 cells, and Vero cells were grown in DMEM medium with 10% heat-inactivated fetal bovine serum (FBS). hCMEC/D3 were grown in EBM2 medium (Clonetics, http://www.cambrex.com) with 5% heat-inactivated FBS. A549 cells were grown in F12K medium with 10% heat-inactivated FBS. TrHBMEC and hCMEC/D3 cells, a kind gift of Babette Weksler, Ignacio Romero, and Pierre-Olivier Couraud, are permanent human endothelial cell lines isolated from the bone marrow and the brain, respectively [32,33]. MRC5 cells are primary human fetal lung epithelial cells (ATCC number CCL-171). A549 (ATCC number CCL-185) are human malignant alveolar type II pneumocytes and BEAS-2B (ATCC number CRL-9609) cells are SV40-transformed human airway epithelial cells. Resting MRC5 cells were obtained by maintaining confluent cultures in serum-free medium with 10^−6^ M dexametasone for at least 10 d before viral exposure (95% of the cells at G0/G1 by flow cytometry analysis of the cell cycle, not shown). Buffy-coat PBMCs from healthy donors were isolated by Ficoll centrifugation. CD4+ T lymphocytes were isolated by negative selection using magnetic beads (Miltenyi Biotec, http://www.miltenyibiotec.com). B-420 cells are EBV-immortalized B cells [62]. THP-1 and U937 monocytoid cells, Jurkat T cells, PBMCs, and primary lymphocytes and monocytes were grown in RPMI medium with 10% FBS. A. albopictus C6/36 cells were grown at 28 °C in Leibovitz-L15 medium supplemented with 5% FBS and tryptose-phosphate. DCs were prepared as described [62,63]. CD14+ monocytes were isolated from PBMCs by positive selection using magnetic beads (Miltenyi Biotec). To allow differentiation of macrophages, monocytes were cultured for 7 d before use in RPMI 1640 medium with 5% human AB-positive serum and 5% SVF and rHu M-CSF (12.5 ng/mL) (Promokine, http://www.promokine.de). Chloroquine and bafilomycin were from Sigma (http://www.sigmaaldrich.com), IFNs from ImmunoTools (http://www.immunotools.de).

### Antibodies.

The following antibodies were used: a mAb directed against a conserved region of the alphavirus nucleocapsid protein (anti-C), a kind gift of Isabelle Greiser-Wilke [28]; mouse polyclonal anti-CHIKV antibodies [5]; anti-Dyn-2 mAb (Affinity BioReagents, http://www.bioreagents.com); anti-actin mAb (Sigma); and anti-active caspase-3 mAb (Caspase 3 Apoptosis Kit; BD Pharmingen, http://www.bdbiosciences.com).

### CHIKV titration.

Viral samples were titrated as TCID50/mL on Vero cells using a standard procedure. Briefly, serial dilutions (100 μL) of cell culture supernatants were added in 8-plicates into 96-well plates seeded with 10^4^ Vero cells. The CPE was scored 5 d after infection and the titer was calculated by determining the last dilution giving 50% of wells with cells displaying a CPE. Similar titers were obtained using a focus immunoassay [5].

### CHIKV infection.

The preparation of CHIKV from clinical samples has been described [5]. The four CHIKV strains were propagated twice in C6/36 cells, and supernatants were harvested and frozen at −80 °C as described [5] before titration and further use. Adherent cells (plated at about 25% confluence in 6- or 24-well plates), and nonadherent cells (0.5 × 10^6^–1 × 10^6^ cells/experimental point) were exposed to the indicated viruses for 2–4 h at 37 °C, extensively washed, and cultivated for various periods of time before further analysis. The moi was defined as the amount of CHIKV infectious units (calculated on Vero cells as a TCID50) per one target cell.

### CHIKV binding.

Adherent cells (plated at about 5 × 10^5^ cells/experimental point in 6-well plates) and nonadherent cells (5 × 10^5^ cells/experimental point) were exposed (1 h at 4 °C) to CHIKV (CHIKV-21 or CHIKV-115 strains) at the indicated moi in a final volume of 1 ml and extensively washed. Expression of CHIKV proteins was then analyzed by flow cytometry after staining with mouse polyclonal anti-CHIKV antibodies.

### Flow cytometry, confocal microscopy, and Western blot analysis.

Expression of CHIKV proteins was analyzed on permeabilized cells with the anti-alphavirus C mAb [28] or with the polyclonal anti-CHIKV serum [5]. Samples were analyzed by flow cytometry using a FACSCalibur (Becton Dickinson, http://www.bdbiosciences.com), with FlowJo software (http://www.flowjo.com). For immunofluorescence, cells were analyzed by confocal microscopy on a Zeiss LSM510 instrument (http://www.zeiss.com). For Western blot, cells were lyzed in lysis buffer (1% Triton X-100–PBS) with Complete Protease Inhibitor Cocktail tablets (Roche, http://www.roche.com). Cell supernatants (1 ml) were pelleted by ultracentrifugation (45 min at 50,000 rpm) in a TL-100 rotor (Beckman, http://www.beckman.com), and resuspended in 100 μl of lysis buffer. An equivalent of 10 μg of total cell lysate or 15 μl of ultracentrifugated supernatant was loaded on each well of a 4%–12 % gradient polyacrylamide gel. Western blotting was performed with a mix of anti-C and anti-CHIKV antibodies.

### Cell viability.

Cell viability was measured using a classical MTT assay [64]. HeLa cells were plated in 96-well plates and exposed to the indicated virus. After 24 to 48 h, 70 μg of MTT (3-(4,5-dimethyl-2-thiazolyl)-2,5-diphenyl-2H-tetrazolium bromide, Sigma) (10 μL of 7 mg/mL solution) was added to 100 μL of culture medium for a 5-h incubation in a CO_2_ incubator at 37 °C. The medium was then removed and crystals were solubilized using 100 μL acid isopropanol (0.04 N HCl in isopropanol). Plates were read on an ELISA reader using a test wavelength of 550 nm.

### Quantification of CHIKV RNA by real-time PCR.

Cells were exposed to the indicated moi of CHIKV for 2–4 h, washed with PBS, and cultured for the indicated periods of time. RNA was prepared from supernatants using the MagNA pure LC total nucleic acid isolation kit (Roche Diagnostics, http://www.roche.com). CHIKV RNA levels were measured with a Light Cycler (Roche Diagnostics) (P. Laurent, K. Le Roux, P. Grivard, G. Bertil, F. Naze, et al., unpublished data).

### Electron microscopy.

HeLa cells were infected with CHIKV (moi 1). At 36 h after infection, cells were fixed in PBS-3% glutaraldehyde for 1 h and post-fixed in PBS-1% osmium tetroxide for 2 h. After rinsing in PBS, cells were transferred to 0.2 M cacodylate buffer for 30 min. Cells were washed in 30% methanol for 10 min, stained in 2% uranyl acetate–30% methanol for 1 h, and washed in 30% methanol. Cells were then dehydrated in an ethanol series to propylene oxyde and embedded in Epon 812. Cells were examined with a JEOL 1200EX2 microscope (http://www.jeol.com).

### Lentiviral vectors expressing shRNAs.

Lentiviral vectors expressing GFP and shRNA (against Dyn-2 or an irrelevant protein as a control) were produced and used as described [39].

## Abbreviations

CHIKV: chikungunya virus
CPE: cytopathic effect
DC: dendritic cell
Dyn-2: dynamin-2
IFN: interferon
mAb: monoclonal antibody
moi: multiplicity of infection
PBMC: peripheral blood mononuclear cell
pi: post-infection
RRV: Ross River virus
SD: standard deviation
SFV: Semliki Forest virus
shRNA: short hairpin RNA
SINV: Sindbis virus
TCID50: tissue culture infectious dose 50
